# Severe Osteolysis of the Mandibular Angle and Total Condylolysis in Progressive Systemic Sclerosis

**DOI:** 10.1155/2013/948042

**Published:** 2013-12-09

**Authors:** Amin Rahpeyma, Seyed Hosein Hoseini Zarch, Saeedeh Khajehahmadi

**Affiliations:** ^1^Oral and Maxillofacial Surgery, Oral and Maxillofacial Diseases Research Center, Faculty of Dentistry, Mashhad University of Medical Sciences, Vakilabad Boulevard, Mashhad, Iran; ^2^Oral and Maxillofacial Radiology, Dental Research Center, Faculty of Dentistry, Mashhad University of Medical Sciences, Vakilabad Boulevard, Mashhad, Iran; ^3^Oral and Maxillofacial Pathology, Dental Research Center, Faculty of Dentistry, Mashhad University of Medical Sciences, Vakilabad Boulevard, P.O. Box 91735-984, Mashhad, Iran

## Abstract

Progressive systemic sclerosis is an autoimmune disease characterized by gradual deposition of abnormal collagen which causes fibrous changes of the skin and visceral organs such as lung, heart, and kidney, which lead to serious complication. Microangiopathy and firm skin cause extrinsic pressure and obliteration of the vessels that lead to ischemia and destruction of the underlying bone. Salivary gland fibrosis causes xerostomia that increases risk of dental caries. Therefore, oral hygiene instruction (OHI) and preventive dentistry are recommended for these patients. We present a 36-year-old female that suffers from systemic sclerosis with total lysis of the mandibular condyle.

## 1. Introduction

Scleroderma or progressive systemic sclerosis (PSS) was first reported in 1752 by Curzio of Naples [1]. PSS, also known as scleroderma, is a connective tissue disorder characterized by fibrosis of the skin and visceral organs [2]. The cause of PSS is immunologically mediated pathogenesis. PSS occurs predominately in the adult but it can also be seen in childhood, with females affected three to four times as often as males [3]. The most common manifestations is microstomia that causes a limitation of opening the mouth in two-thirds of the cases. In addition, salivary gland fibrosis causes xerostomia; therefore, these patients are more prone to dental decay.

In some patients, as loss of attachment gingival occurs, multiple area of gingival recession may be present. In scleroderma patients, preventive care is of foremost importance. Meticulous oral hygiene must be established and maintained. Occasionally slight bilateral erosion of mandibular condyle causes anterior open bite. Bone resorption is caused by the increased pressure associated with the deposition of collagen.

Bone resorption has been reported in maxillofacial region, which occurs in masticatory muscle attachments [4]. On radiographic appearance a characteristic resorption of the ramus and condyle may be observed.

We reported a case of total osteolysis of the mandibular condyle in a 36-year-old female patient with oral manifestations of the disease.

## 2. Case Report

A 36-year-old female suffering from PSS with 9-year history of disease was referred to oral and maxillofacial surgery for extraction of her decayed posterior teeth.

The patient had reduced maximal interincisal opening (MIO = 12 mm), mask-like face, perioral deep rhytides, pinched nose, facial telangiectasia, and atrophic ulcerated finger tips (sclerodactyly). Pain accompanying bone resorption is present in her fingers (painful digital ischemia).

**Figure 1:**
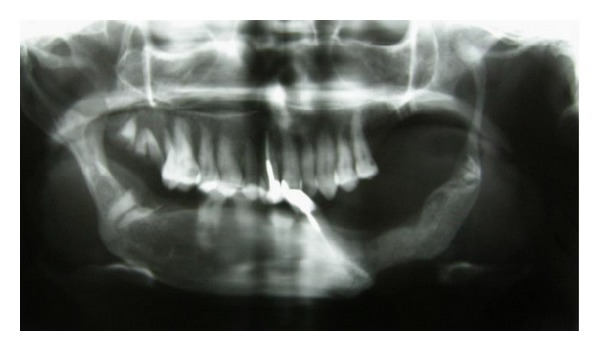
Dental panoramic tomogram relieved extensive bone resorption of bilateral mandibular angles and total left condylolysis.

**Figure 2:**
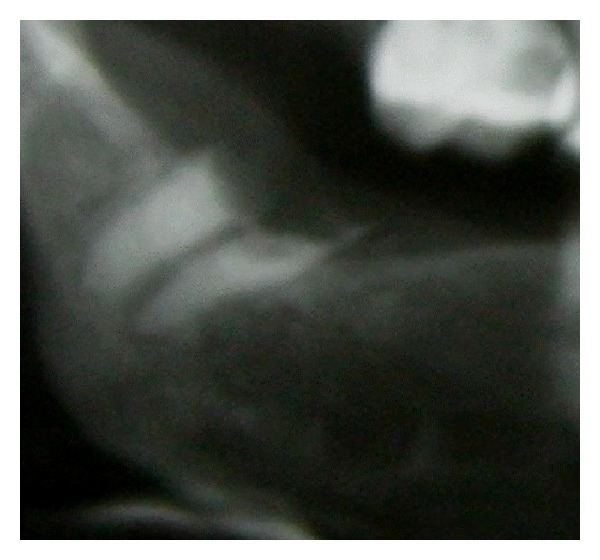
Resorption of the right mandibular angle and decayed remnant root tooth.

In medical history, there was pulmonary fibrosis. Limitation in mouth opening, malformed fingers, dry mouth, tight tongue, and reduced movement of the cheeks created problems in oral hygiene maintenance; hence molar teeth were severely decayed. Root canal therapy (RCT) of teeth number 9 and number 22 with pin composite buildup was observed (Figure 1). Decayed remained roots were present in posterior quadrants (Figure 2). Dental treatment such as posterior teeth restorations, fixed or removable prosthesis, was difficult to do in this patient because of restricted mouth opening.

**Figure 3:**
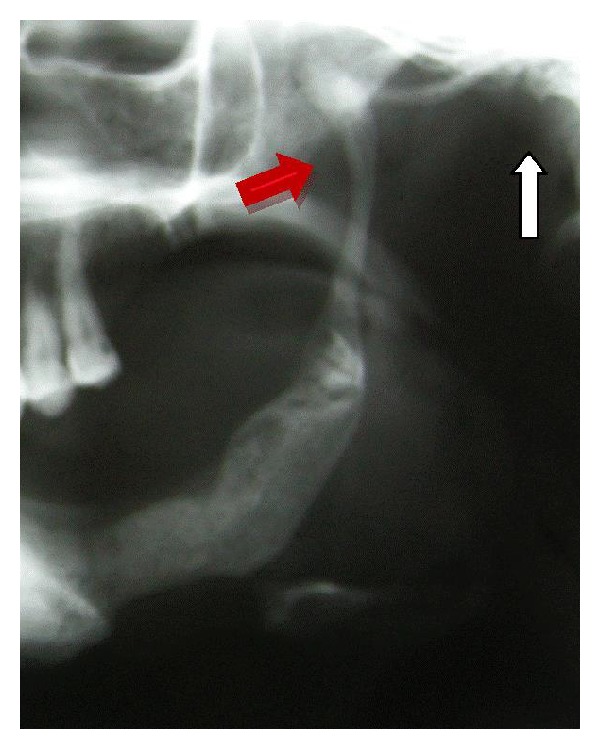
Empty glenoid fossa (white arrow), thin elongated coronoid process (red arrow), and left angle bone resorption.

Dental panoramic view relieved extensive bone resorption of bilateral mandibular angles and total left condylolysis (Figure 3). Left coronoid process was thinned and elongated which extended up to zygomatic arch. Contralateral coronoid process length was normal. There was a 1.5 cm vertical length difference between right and left coronoid processes. Patient has no history of jaw trauma or surgery.

Under local anesthesia, decayed roots were removed. Limitation in mouth opening and severe bone resorption made dental procedures difficult. Great caution is required to avoid iatrogenic jaw fracture.

## 3. Discussion

Scleroderma or PSS is an autoimmune disease characterized by gradual deposition of collagen in reticular dermis, gastrointestinal tract, heart, lung, and other organs [2]. most cases of PSS most commonly occur during the third to fifth decades of life [2]. PSS has a low prevalence (130 per million) [3] and it occurs about three times more common in women.

Bone resorption is an uncommon occurrence but recognized complication of long standing PSS [5]. Posterior inferior ramal bone resorption of the jaws was first described in 1959 by Taversa [6]. It is hypothesized that pressure from overlying skin and atrophic muscles with ischemia from small vessels angiopathy has role in bone resorption pathogenesis [7]. In maxillofacial regions resorption occurs in attachment area of masticatory muscles including masseter, temporal, lateral pterygoid, and anterior belly of digastrics muscle. Bone resorption in other skeletal bones such as terminal phalanges, distal radius, ulna, cervical spine, zygomatic arch, and the ribs has been reported [8].

Mandibular bone resorption usually happens in mandibular angle area and is more likely to happen bilaterally [8–10].

Pervious reported cases of bilateral mandibular condylolysis in scleroderma patients had open bite except the case of Caplan [11–13]. Despite extensive resorption of the condyle, the present case had no open bite or alteration in jaw relationship.

Pain, preauricular tenderness, occlusal disturbance, TMJ sounds, and reduction of ramus height are common clinical features, but in our case despite huge bone resorption, there were no pain and tenderness. Dental radiographic manifestations of the scleroderma patient include PDL widening, root resorption, and decayed posterior teeth [14] similar to our case. Patients with PSS exhibit an increased prevalence of external root resorption [5].

Root canal therapies of the teeth with external root resorption require special consideration.

Fibrosis of the salivary glands leads to xerostomia [15]. Because of dry mouth, these patients have a greater risk for dental caries and periodontal disease and in this case we should consider concurrent secondary sjogren syndrome. In addition, there is an increased prevalence of oral candidacies in patients with xerostomia because of reduction in saliva production.

In the present case, dry mouth was an important clinical feature; thus preventive dentistry is too important in scleroderma patients.

Also diffuse widening of the periodontal ligament space is often present in dental radiograph.

In our case, despite complete left mandibular condylolysis and bilateral angle resorption, there were appropriate jaw relations.

Perimandibular connective tissue (PMCT) adaptation and presence of mandibular canines help in stabilization of three dimensional spatial positions of jaws.

Limited mouth opening and reduced movement of the cheek and tongue made dental practice very difficult. Dental treatment including removable partial dentures, traditional fixed prosthodontics, or implant is difficult [5]. In order to reduce dental problems, programmed preventive dentistry and regular followup of the scleroderma patients are recommended.

Deposition of abnormal collagen in the tongue causes a firm and hypomobile tongue [5]. The oral chief compliant in scleroderma patients is xerostomia, which causes difficulty in speaking. Some patients who complain of dry mouth may appear to have adequate salivary flow and oral moistness.

## 4. Conclusion

Because of the increased potential for dental caries in scleroderma patient, programmed preventive dentistry and regular followup are recommended.

Reduced maximal interincisal opening and severe bone resorption made dental procedures difficult. Great caution is required for avoiding iatrogenic jaw fracture in severely resorbed mandibular bone.
